# Assessing seizure liability *in vitro* with voltage-sensitive dye imaging in mouse hippocampal slices

**DOI:** 10.3389/fncel.2023.1217368

**Published:** 2023-08-23

**Authors:** Yuichi Utsumi, Makiko Taketoshi, Michiko Miwa, Yoko Tominaga, Takashi Tominaga

**Affiliations:** ^1^Graduate School of Pharmaceutical Sciences, Tokushima Bunri University, Sanuki, Japan; ^2^Institute of Neuroscience, Tokushima Bunri University, Sanuki, Japan; ^3^Kagawa School of Pharmaceutical Sciences, Tokushima Bunri University, Sanuki, Japan

**Keywords:** voltage-sensitive dye, hippocampus, seizure liability, optical recording, stratum radiatum, stratum pyramidale, drug-induced seizures, toxicology

## Abstract

Non-clinical toxicology is a major cause of drug candidate attrition during development. In particular, drug-induced seizures are the most common finding in central nervous system (CNS) toxicity. Current safety pharmacology tests for assessing CNS functions are often inadequate in detecting seizure-inducing compounds early in drug development, leading to significant delays. This paper presents an *in vitro* seizure liability assay using voltage-sensitive dye (VSD) imaging techniques in hippocampal brain slices, offering a powerful alternative to traditional electrophysiological methods. Hippocampal slices were isolated from mice, and VSD optical responses evoked by stimulating the Schaffer collateral pathway were recorded and analyzed in the stratum radiatum (SR) and stratum pyramidale (SP). VSDs allow for the comprehensive visualization of neuronal action potentials and postsynaptic potentials on a millisecond timescale. By employing this approach, we investigated the *in vitro* drug-induced seizure liability of representative pro-convulsant compounds. Picrotoxin (PiTX; 1–100 μM), gabazine (GZ; 0.1–10 μM), and 4-aminopyridine (4AP; 10–100 μM) exhibited seizure-like responses in the hippocampus, but pilocarpine hydrochloride (Pilo; 10–100 μM) did not. Our findings demonstrate the potential of VSD-based assays in identifying seizurogenic compounds during early drug discovery, thereby reducing delays in drug development and providing insights into the mechanisms underlying seizure induction and the associated risks of pro-convulsant compounds.

## 1. Introduction

Non-clinical toxicity is a leading cause of drug candidate attrition during drug development (Waring et al., 2015). To address this issue, regulatory agencies, such as the International Council for Harmonisation (ICH), have developed guidelines for safety pharmacology testing that require the evaluation of cardiovascular, respiratory, and central nervous system (CNS) functions prior to human administration. Specifically, the ICH guideline S7A mandates safety pharmacology tests including those that evaluate CNS function. The Irwin test (Irwin, 1968) and functional observational battery (Moser et al., 1995) have been frequently used in preclinical safety studies to detect the potential toxicity of drugs on the CNS. Of the potential CNS toxicities detected through these tests, the most common finding is drug-induced seizures (Authier et al., 2016a). Although electroencephalogram (EEG) analysis can also detect seizures in laboratory animals, pre- or post-dose non-invasive EEG assessments are not commonly included in these studies (Authier et al., 2016b). As a result, drug-induced seizures are often not identified until later stages of drug development, which can cause significant delays and negatively impact the timeline of drug development. Therefore, an *in vitro* seizure liability assay developed and implemented in early drug discovery could help identify potential seizure-inducing compounds and reduce delays in drug development.

A seizure is a complex process involving multiple cellular mechanisms and therefore multiple pharmacological targets. *In vitro* brain slices, which retain many of the neural circuits and signaling pathways present in the brain, have been used as a model system for CNS activity in early drug development and safety studies (Easter et al., 2007, 2009; Accardi et al., 2016). The hippocampus, in particular, is known to play a crucial role in seizure induction (Schwartzkroin, 1994). Recently, seizure liability assessments using hippocampal brain slices from different animal species, such as rats, dogs, monkeys, and mini-pigs, have been performed and found to be sensitive to pro- and anti-convulsant agents (Accardi et al., 2018). Currently, besides whole animal behavior tests, hippocampal brain slice electrophysiology tests are considered the standard for seizure-liability testing (Easter et al., 2007; Accardi et al., 2018; Zhai et al., 2021).

Traditional electrophysiological techniques are limited by their inability to increase the number of electrodes and measure individual cell activity, leading to challenges in comprehensively monitoring neural activity. However, voltage-sensitive dyes (VSDs) can be incorporated into nerve cell membranes to produce optical signals in response to changes in nerve membrane potential, providing a valuable alternative to traditional electrophysiological techniques (Cohen et al., 1978; Cohen and Salzberg, 1978; Tominaga et al., 2000, 2013, 2023; Grinvald and Hildesheim, 2004; Peterka et al., 2011). VSDs allow for the optical measurement of membrane potential changes in milliseconds, providing a comprehensive visualization of neuronal action potentials and synaptic potentials. Recent advances in VSD imaging methods have enabled the measurement of subtle changes in neural circuit function, including those associated with gene-manipulated animals (Tanemura et al., 2002; Suh et al., 2011), developmental modifications induced by drugs (Juliandi et al., 2015; Ishihara et al., 2022), and diseases (Mann et al., 2005; Hayase et al., 2020). In this study, we demonstrated that the use of VSD-based assays to quantify and visualize neural circuit function in hippocampal slices is critical for assessing the *in vitro* drug-induced seizure liability of representative pro-convulsant compounds, such as picrotoxin (PiTX), gabazine (GZ), 4-aminopyridine (4AP), and pilocarpine hydrochloride (Pilo). Our results indicate that VSD methods can discriminate layer-specific effects of modulation by these compounds, as evidenced by contrasting the signal from the cell layer (stratum pyramidale) with that from the dendritic membrane potential response (stratum radiatum). Moreover, the manner in which dendritic membrane potential signals vary depends on the pharmacological effects of the compounds on membrane potential modulation. These results highlight the potential advantages of VSD spatial resolution and population intracellular signal over conventional field potential recordings.

## 2. Materials and methods

### 2.1. Slice preparation and staining with VSD

All animal experiments were performed according to protocols approved by the Animal Care and Use Committee of Tokushima Bunri University. Hippocampal slices (350 μm thick) were prepared from 4- to 7-week-old male mice (C57BL6) who were decapitated under deep isoflurane anesthesia. The preparation and staining of the VSD are the same as that in the method published by Tominaga et al. (2019). Briefly, the brains were rapidly cooled in ice-cold artificial cerebrospinal fluid (ACSF) containing 124 mM NaCl, 2.5 mM KCl, 2 mM CaCl_2_, 2 mM MgSO4, 1.25 mM NaH_2_PO_4_, 26 mM NaHCO_3_, and 10 mM glucose, at a pH of 7.4, equilibrated with 95%/5% O_2_/CO_2_ mixed gas. After cooling for 5 min, the hippocampus was dissected along with the surrounding cortex, and the entire hippocampal structure was placed on an agar block. The ventral side of the hippocampus was cut vertically, and this side was attached to a vibratome (VT-1000 and VT-1200S, Leica) with cyanoacrylate glue. Transverse sections (350 μm thick) containing the hippocampus were cut, and each slice was transferred to a fine mesh membrane filter (Omnipore, JHWP01300, 0.45 μm pores, Merck Millipore Ltd., MA, USA) and fixed with a plexiglass ring (11 mm i.d, 15 mm o.d., and 1–2 mm thick). It was then transferred to a moist chamber, whose atmosphere was maintained by 95%/5% O_2_/CO_2_ mixed gas continuously bubbling through the ACSF. The temperature of the chamber was maintained at 28°C for 25 min and then allowed to return to room temperature. After 1 h of incubation, the slices were stained for 20 min with 100–110 μL of VSD staining solution [0.1 mM Di-4-ANEPPS (D-1199, Thermo Fisher Scientific Inc., MA, USA), 2.7% ethanol, 0.13% Cremophor EL (Sigma-Aldrich Co.), 50% fetal bovine serum (Sigma-Aldrich Co.), and 50% ACSF]. Slices were incubated for at least 1 h at room temperature with protection from light prior to recording.

### 2.2. Stimulation and electrophysiological recordings

Hippocampal slices were transferred to a submerged chamber using the plexiglass ring and continuously perfused with ACSF at a rate of 1 mL/min, heated to 31°C, and bubbled with 95%/5% O_2_/CO_2_ mixed gas. Glass electrodes filled with ACSF and inserted with Ag/AgCl wire were used as stimulating and recording electrodes to measure field excitatory postsynaptic potential (fEPSP) in the Schaffer collateral (SC) pathway and the stratum radiatum (SR) of cornu ammonis 1 (CA1). Electrical artifacts were removed from the traces as shown in the results. The stimulation frequency of 0.05 Hz was maintained throughout the experiment. The stimulation intensity was altered using an electrical stimulator (ESTM-8, Brainvision, Inc., Tokyo, Japan) and the IgorPro (WaveMetrics Inc., OR, USA) macro program. Field potential recordings were obtained using a differential amplifier (model 3000; AM Systems, WA, USA; low-pass filtered at 3 kHz, high-pass filter at 0.1 Hz, gain × 100), and digitized by analog inputs of ESTM-8 at 10 kHz sampling (an AD converter of 16 bits) and fed into a computer. The analysis of electrophysiological data was done for these recordings. The electrophysiological and optical recordings did not interfere with one another.

### 2.3. Optical recording with VSD signals

Optical recording of VSD signals was performed concurrently with electrophysiological recordings. Epifluorescence optics consisting of a focus length = 20 mm objective lens (numerical aperture = 0.35; Brainvision Inc., Tokyo, Japan), a × 1.0 Leica Microsystems (as a projection lens), a dichroic mirror (575 nm), and excitation (530 ± 30 nm) and emission (>590 nm) filters were mounted above the slice. Fluorescence was measured and projected onto a CMOS camera (MiCAM02, Brainvision Inc., Tokyo, Japan). The ratio of the fractional change in VSD fluorescence to the initial amount of fluorescence (ΔF/F) was used as the optical signal. The frame rate was 0.6 ms/frame on the MiCAM02 camera (12 bit ADC, 4.5 × 10^5^ well depth, 70 dB). The optical signals presented in the following sections were spatially and temporally filtered twice with a Gaussian kernel of 5 × 5 × 3 (horizontal × vertical × temporal; σ≈ 1). The analysis of the optical signals was performed using Igor Pro software (WaveMetrics Inc., OR, USA). Field potential recordings were also captured with an analog input in the MiCAM02 system, and the time correlation of the electrophysiological data and optical signals was continually confirmed.

### 2.4. Drugs and solutions

The reference compounds used in this study were PiTX, SR95531 (GZ), 4AP, and Pilo (Table 1), which were obtained from Sigma-Aldrich Co. and Tocris. Stock solutions (1000 ×) of each compound were prepared in their respective solvents, aliquoted, and frozen at −20°C. They were then diluted in oxygenated ACSF immediately prior to use. Other reagents used were obtained from distributors in Japan.

**TABLE 1:** Compound reference set including solvent used and reported mode(s) of action.

Compound	Solvent	Description	Concentration (μM)	References
Picrotoxin	Ethanol	GABA_A_ receptor antagonist, used in research to induce seizures	1, 10, 100	Mackenzie et al., 2002
Gabazine	Water	SR95531 is GABA_A_ receptor antagonist, used in research to induce seizures	0.1, 1, 10	Lindquist et al., 2005; Johnston, 2013
4-Aminopyridine	DMSO	Potassium channel blocker, used to induce seizures in *in vivo* experiments	10, 40, 100	Peña and Tapia, 2000
Pilocarpine hydrochloride	Water	Muscarinic acetylcholine (ACh) receptor agonist, used as *in vivo* model for epilepsy	10, 30, 100	Zimmerman, 1981; Marchi et al., 2007

GABA, gamma-aminobutyric acid.

### 2.5. Data and statistical analysis

**FIGURE 1:**
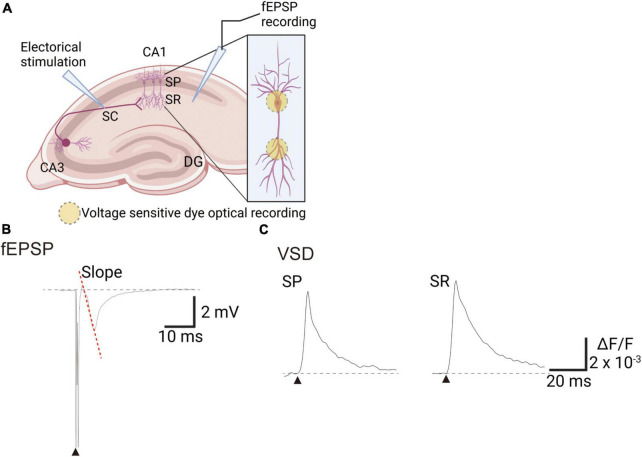
Experimental scheme of recording voltage sensitive dye (VSD) and electrophysiological signals in mice hippocampal slices. **(A)** An illustration showing the arrangement of recording optical and electrophysiological signals. **(B)** Trace of field excitatory postsynaptic potentials (fEPSPs) recorded with the field electrode in the SR. **(C)** Trace of VSD optical signals in the SP or SR. Filled black triangles indicate electrical stimulation. Image created on BioRender.com. DG, dentate gyrus; SC, Schaffer collateral; SP, stratum pyramidale; SR, stratum radiatum; ΔF/F, ratio of the fractional change in VSD fluorescence to the initial amount of fluorescence.

During electrophysiological recordings, field potentials were obtained every 30 s (total 204 times) through electrodes placed in the SR, and the fEPSP slope was calculated (Figures 1A, B). Simultaneously, VSD optical data were recorded every 30 s (0.6 ms/frame × 512 frames, total 307.2 ms) to obtain response waveforms of the SR and stratum pyramidale (SP) (Figures 1A, C). Optical and electrophysiological signals were analyzed concurrently using custom macros developed in Igor Pro software (WaveMetrics Inc., OR, USA). All data are presented as means ± standard error of the mean (SEM), with n representing the number of slices. Statistical analyses were performed using the R statistical software package (4.1.2; R Core Team, 2021). Statistical significance was determined by one-way analysis of variance followed by Dunnett’s test, with a *p*-value less than 0.05 considered significant (*p < 0.05, ^**^p < 0.01, and ^***^p < 0.001).

## 3. Results

### 3.1. Effects of the seizurogenic compounds on the fEPSP slope

**FIGURE 2:**
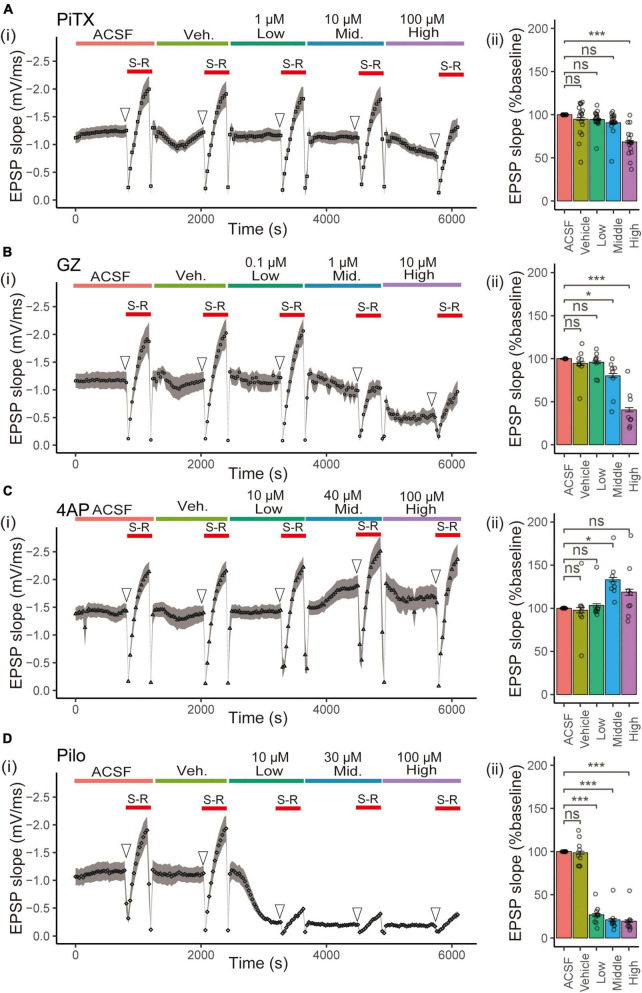
Effect of seizurogenic compounds on fEPSP slope in stratum radiatum. (i) The time courses of the slope of the fEPSP after application of ACSF, vehicle (Veh), and each compound [**(A)** picrotoxin (PiTX), **(B)** gabazine (GZ), **(C)** 4-amino pyridine (4AP), and **(D)** pilocarpine (Pilo)] over a series of three ascending concentrations [Low, Mid (middle), High] for a period of 20 min per concentration. The stimulus -response S, R relationship was done in each concentration of the test compound (red solid line). (ii) Mean data summary of compound effects on EPSP slopes. The mean fEPSP slopes were determined for 10 data points of the fEPSP slopes (open arrowhead) before (S-R) relationship. All error bars, ± SEM from *n* = 8 to 16 slices. *p < 0.05; ***p < 0.001; ns, not significant (Dunnett’s test). fEPSPs, field excitatory postsynaptic potentials; EPSP, excitatory postsynaptic potentials; Mid., middle.

We investigated the effects of four reference compounds, namely PiTX, GZ, 4AP, and Pilo, on fEPSP slope and VSD optical signals in hippocampal slices (Figure 1) by testing three concentrations of each compound. fEPSP is essential as a direct synaptic response to neuronal inputs. In this study, it was used for monitoring the physiological response of the area CA1. The experimental sequence began with a 20 min baseline period in normal ACSF, followed by a test of the stimulus-response (S-R) relationship. Next, the solution was changed to ACSF containing vehicle medium for 20 min, followed by a second S-R test. Three different concentrations of each compound (Table 1) were then tested for 20 min (Figures 2Ai–Di), with concurrent optical recordings made throughout. The VSD optical signals were acquired every 30 s. Therefore, 204 recordings of 307.2 ms were acquired from the same brain slice.

We calculated the mean fEPSP slope from the average of 10 data points of fEPSP slope before the S-R relationship for each treatment, with ACSF as 100% relative to vehicle or each concentration (Figures 2Aii–Dii). PiTX significantly reduced the fEPSP slope at high concentrations (100 μM) (68.63 ± 1.39%, *p* < 0.001), as did GZ at middle concentrations (≥1 μM) (80.39 ± 2.43%, *p* < 0.05; 40.94 ± 2.46%, *p* < 0.001). Pilo strongly reduced the fEPSP slope at low concentrations (≥10 μM) (26.80 ± 1.47%, 21.02 ± 1.66%, 19.70 ± 1.43%, *p* < 0.001), while 4AP significantly raised the fEPSP slope at the middle concentrations (40 μM) (133.16 ± 2.39%, *p* < 0.05), it tended to raise its slope at high concentrations (100 μM).

### 3.2. Effect of seizurogenic compounds on VSD imaging in hippocampal slice

**FIGURE 3:**
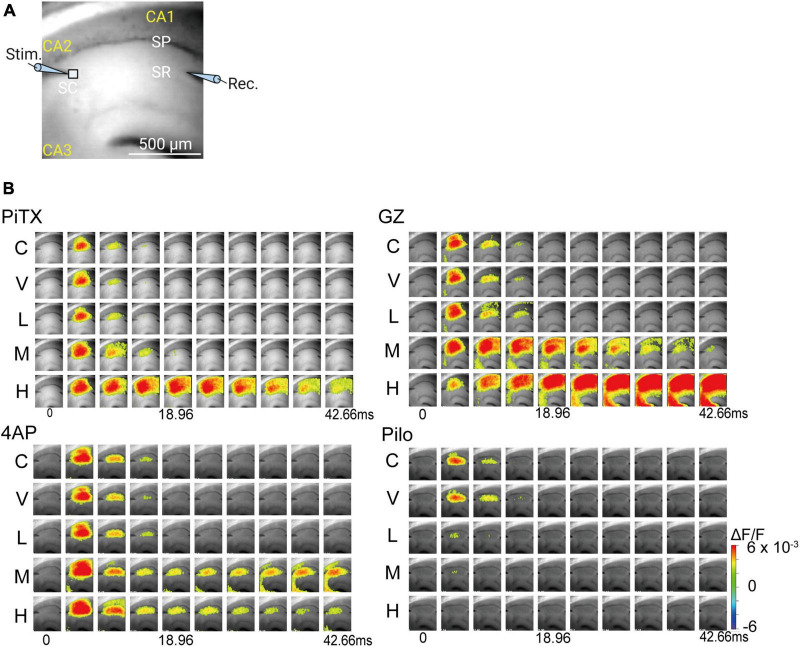
Spread of neural activity upon single stimulation of hippocampus under seizurogenic compounds treatment. **(A)** Fluorescent image of mouse hippocampal slices captured with 90 × 80 pixels high speed camera system. **(B)** Typical spread pattern of evoked neural activity following electrical stimulation. The sequence of images are the neural activity map after stimulation with: PiTX (upper left), GZ (upper right), 4AP (lower left), and Pilo (lower right). Depolarization was measured as fractional changes in fluorescence in each pixel; this value is encoded in pseudocolor as indicated in the scale and superimposed on a fluorescent image of the slice. PiTX, picrotoxin; GZ, gabazine; 4AP, 4-amino pyridine; Pilo, pilocarpine; C, control; V, vehicle; L, low; M, middle; H, high; ΔF/F, ratio of the fractional change in voltage-sensitive dye fluorescence to the initial amount of fluorescence.

We used VSD imaging to observe the spread pattern of neuronal activity in response to Schaffer collateral pathway stimulation. A bright field view of the brain slice in the observation area is shown in Figure 3A. PiTX induced expanded neuronal activity area in the CA1 region and prolonged propagation at the middle concentration (10 μM), with marked effects at high concentrations (100 μM) (Figure 3B, upper left). Similarly, GZ induced significantly expanded neuronal activity area in the CA1 region with prolonged propagation and propagation to the CA3 area at middle concentrations (≥1 μM) (Figure 3B, upper right). 4AP also induced significantly expanded neuronal activity area in the CA1 region with prolonged propagation and propagation to the CA3 area at middle concentrations (≥40 μM) (Figure 3B, lower left). In contrast, Pilo caused a marked reduction in the propagation area in the CA1 region and shortened propagation at low concentrations (≥10 μM) (Figure 3B, lower right).

### 3.3. Effect of seizurogenic compounds on VSD optical response

**FIGURE 4:**
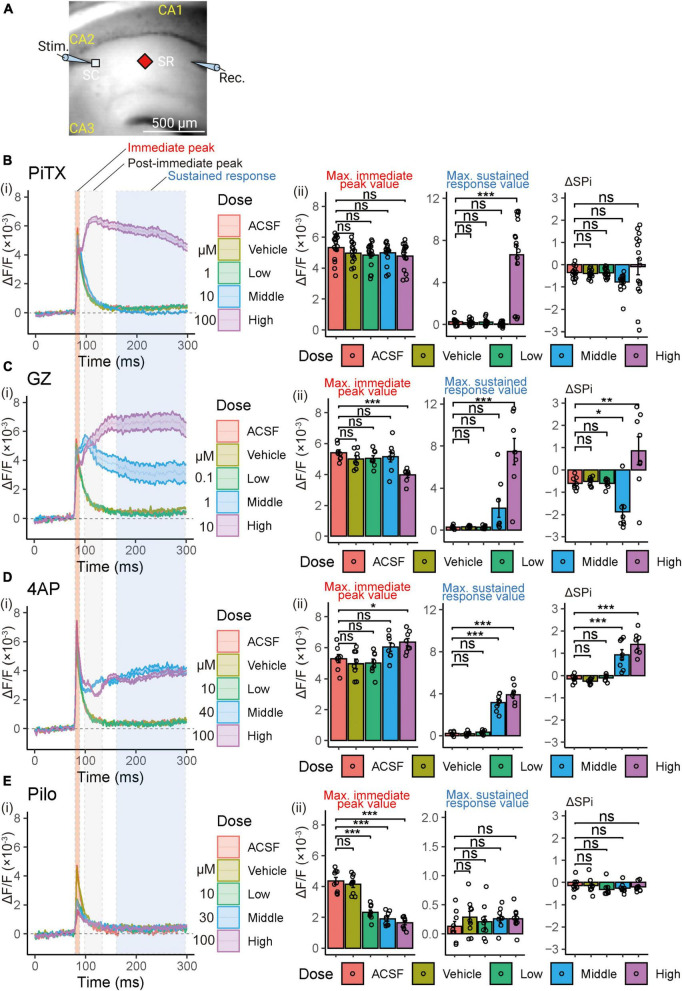
Voltage-sensitive dye (VSD) optical response waveforms analysis following single stimulation in SR. **(A)** Illustration of the recording setup for optical signal detection (Captured 90 × 80 pixels high speed camera system). Maximum values of 3 parameter (immediate peak, post-immediate peak, and sustained response) were utilized for the analysis. Figure created using BioRender.com. **(B–E)** (i) Comparison of alterations in VSD optical response waveforms. Solid lines and corresponding shaded areas represent mean ± SEM, derived from *n* = 8 to 16 slices. (ii) Comparison of the maximum immediate peak values of the VSD optical response in the presence of increasing compound concentrations (left panel). Comparison of the maximum sustained response values of the VSD optical response in the presence of increasing compound concentrations (center panel). Comparison of the delta maximum values of the VSD optical response (sustained response value—post-immediate peak value; ΔSPi) in the presence of increasing compound concentrations (right panel). Effects are depicted as ΔF/F ± SEM from *n* = 8 to 16 slices. *p < 0.05; **p < 0.01; ***p < 0.001; ns, not significant (Dunnett’s test). ACSF, artificial cerebrospinal fluid; PiTX, picrotoxin; GZ, gabazine; 4AP, 4-amino pyridine; Pilo, pilocarpine; VSD, voltage-sensitive dye; SC, Schaffer collateral; SP, stratum pyramidale; SR, stratum radiatum.

To further analyze the VSD signal, we compared waveforms of the optical signal at the middle of the SR to examine the effects of each compound. Figure 4A illustrates the arrangement of recording optical signals. All compounds caused changes in the waveform. PiTX at high concentrations (100 μM) and GZ at middle and high concentrations (1 and 10 μM) showed an increase in sustained response (Figures 4Bi, Ci). 4AP showed a clear increase in the immediate peak value at high concentrations (100 μM), and an increase in sustained response at middle or higher concentrations (≥40 μM) (Figure 4Di). However, Pilo showed a distinct decrease in the amplitude of the immediate peak (Figure 4Ei).

### 3.4. Comparison of defined parameters in VSD optical response

To elucidate the mechanisms underlying the effects of reference compounds, we further analyzed the optical traces by evaluating three parameters: the immediate peak value, which corresponds to the excitatory postsynaptic potential (EPSP); the post-immediate peak value, defined as the signal size between 50 and 100 ms, where the feedback inhibitory response typically appears; and the sustained response, corresponding to the signal size after 150 ms from stimulation, which reflects the sustained response characteristic induced by the reference compounds (Figures 4Bi–Ei).

We calculated the maximum values for the immediate peak, post-immediate peak, and sustained responses (Figures 4Bii–Eii) for quantitative analysis of waveform data (averaging 10 waveforms following 20 min exposure to the drugs). PiTX did not impact the immediate peak but increased the sustained peak (6.65 ± 0.97, *p* < 0.001) at a high concentration (100 μM). GZ reduced the immediate peak (3.97 ± 0.15, *p* < 0.001) and elevated the sustained peak (7.47 ± 1.24, *p* < 0.001) at a high concentration (10 μM). 4AP also increased the immediate peak (6.36 ± 0.24, *p* < 0.05) at a high concentration (100 μM) and elevated the sustained peak at both middle and high concentrations (40 and 100 μM) (3.17 ± 0.28, 3.91 ± 0.33, respectively; *p* < 0.001 each). In contrast, Pilo decreased the immediate peak at low, middle, and high concentrations (10 to 100 μM) (2.32 ± 0.15, 1.90 ± 0.14, 1.65 ± 0.14, respectively; *p* < 0.001 each) but did not affect the sustained peak value.

Moreover, we calculated the difference between the maximum sustained response value and the maximum post-immediate peak value as ΔSPi (sustained response value minus post-immediate peak value) (Figures 4Bii–Eii). Neither PiTX nor Pilo influenced ΔSPi, while GZ reduced ΔSPi (−1.87 ± 0.32, *p* < 0.05) at a middle concentration (1 μM) and increased ΔSPi (0.86 ± 0.62, *p* < 0.01) at a high concentration (10 μM). 4AP also elevated ΔSPi at middle and high concentrations (40 and 100 μM) (0.93 ± 0.23, 1.40 ± 0.18, respectively; *p* < 0.001 each).

### 3.5. Effect of seizurogenic compounds on the ratio of VSD optical response between stratum pyramidale and SR (PR-ratio)

**FIGURE 5:**
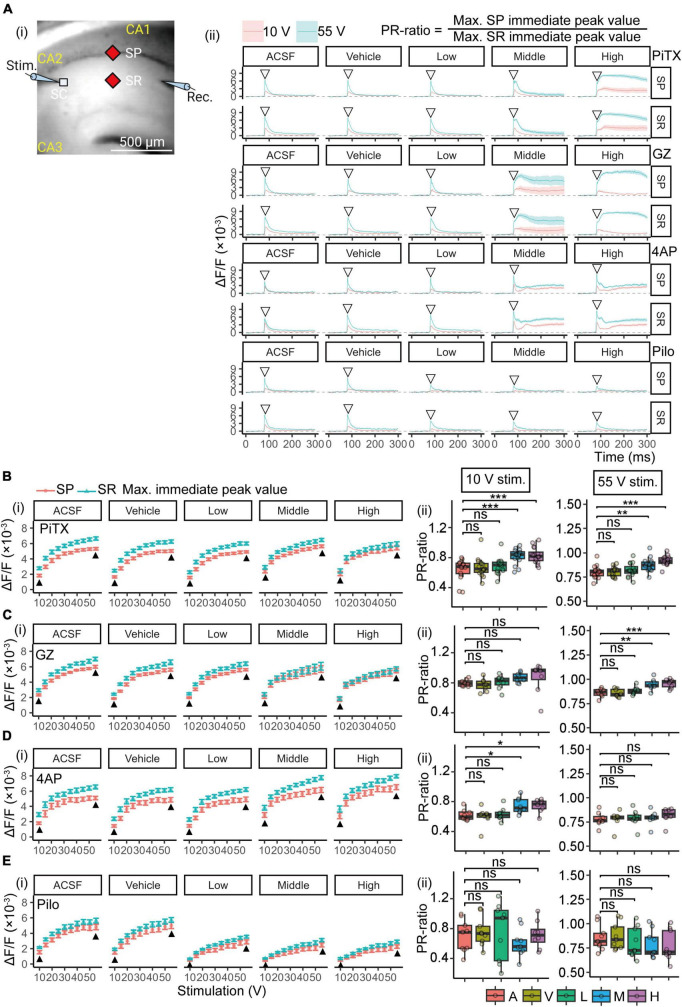
Voltage-sensitive dye (VSD) optical response analysis for E-S coupling. **(A)** An illustration demonstrating the arrangement for recording optical signals with increment stimulation of 10- to 55-volt. Measurement points of optical signals were shown filled red diamond in the **(A)** (i) image. The maximum values of SP and SR immediate peaks [open invert-triangles in the image of **(A)** (ii)] are represented. The PR-ratio is defined as the ratio of its values of SP and SR immediate peaks [**(A)** (ii), right]. **(B–E)** (i) Changes in the maximum immediate peak value of SP and SR are shown at each concentration and stimulus intensity. Black filled triangles indicate maximum immediate peak values of 10- and 55-volt. Plots are illustrated as mean ± SEM from *n* = 8 to 16 slices. (ii) Comparison of the PR-ratio in weak (10-volt) and saturated (55-volt) stimulation. Boxplot of PR-ratios are illustrated from *n* = 8 to 16 slices. *p < 0.05; **p < 0.01; ***p < 0.001; ns, not significant (Dunnett’s test). VSD, voltage-sensitivity dye; PiTX, picrotoxin; GZ, gabazine; 4AP, 4-amino pyridine; Pilo, pilocarpine; ΔF/F, ratio of the fractional change in voltage-sensitive dye fluorescence to the initial amount of fluorescence; SR, stratum radiatum; SP, stratum pyramidale; C, control; V, vehicle; L, low; M, middle; H, high.

Waveforms of the VSD optical response in the SR and SP were obtained as described in section “3.1. Effects of the seizurogenic compounds on the fEPSP slope.” The optical signal in the SR is dependent on EPSP, while that in the SR is dependent on spike occurrence (Tominaga et al., 2009). Consequently, the ratio of immediate peak values in the SR and SP indicates the excitation-spike (E-S) firing relationship. Therefore, we employed the ratio of the SP response to the SR response (PR-ratio) as a measure of the E-S relationship’s strength. Maximum peak values were calculated from the VSD optical response data of the stimulus-response relationship (Figures 2Ai–Di, 5A; red solid line) for each concentration of treatment. The concentration and treatment of each compound were consistent with those in section “3.1. Effects of the seizurogenic compounds on the fEPSP slope.” Representative VSD optical response waveforms (10- and 55-volt stimulation) of each compound are shown in Figure 5Aii. These waveforms showed similar changes to those in Figures 4Bi–Ei.

The PR-ratio is a measure of the excitatory-inhibitory ratio and is sensitive to the inhibitory input to the soma, particularly at weak stimulation levels (Tominaga et al., 2009). Figures 5Bi–Ei show the changes in the maximum immediate peak value of SP and SR, which is necessary for the calculation of PR-ratio, by each concentration and stimulus intensity. The maximum values in SP and SR of PiTX and GZ did not change with increasing concentration (Figures 5Bi, Ci). 4AP produced a concentration-dependent increase in the maximum values of SP and SR (Figure 5Di), while Pilo caused a concentration-dependent decrease (Figure 5Ei). The PR-ratio for the optical signal was calculated as the ratio of the maximum immediate peak value of SP and that of SR in each measurement point (Figure 5Ai, filled red diamond). Figures 5Bii–5Eii represent the comparison of the PR-ratio, calculated for weak (10 V) and saturation (55 V) stimuli, respectively. PiTX showed a significant increase in the average PR-ratio at middle or higher concentrations (≥10 μM) for both 10- and 55-V stimuli (Figure 5Bii). Similarly, GZ displayed a tendency to elevate the average PR-ratio at middle or higher concentrations (≥1 μM), with a significant difference observed only at 55-V stimulation (Figure 5Cii). These findings suggest a reduction in inhibitory action at the soma caused by PiTX and GZ. In contrast, 4AP exhibited an increase in the average PR-ratio at middle or higher concentrations (≥40 μM), but this effect was limited to 10-V stimulation (Figure 5Dii), indicating minimal impact on the E-S relationship. On the other hand, Pilo demonstrated a decreasing trend in the average PR-ratio at middle and low or higher concentrations (30 and ≥10 μM) for weak and saturation stimuli, respectively. However, no significant differences were observed at either 10- or 55-V stimuli (Figure 5Eii). Therefore, PiTX and GZ exhibited a clear reduction in inhibitory action at the soma, leading to a noticeable increase in the PR-ratio. Meanwhile, 4AP had only a modest effect on the E-S relationship, and Pilo had little influence on the PR-ratio and did not show statistical significance for the chosen stimuli.

## 4. Discussion

*In vitro* brain slice assays, such as electrophysiological assays using hippocampal slices for seizure liability evaluation, have been employed in early safety pharmacology assessments (Easter et al., 2009; Accardi et al., 2016). Despite the relatively high predictive rate of 89% for this assay (Easter et al., 2009), differences in responses have been observed between rodents and humans (Löscher, 2009; Bankstahl et al., 2012; Nagayama, 2015). However, VSD-based assays can measure membrane potential changes in milliseconds, enabling comprehensive visualization of neuronal action potentials and synaptic receptive potentials (Tominaga et al., 2009). Leveraging this capability, our study examined the effects of four compounds known to induce seizures in animal models and humans on electrophysiological parameters (fEPSP slope) and VSD optical response. To the best of our knowledge, this study represents the first application of seizure liability assessment using a VSD-based assay *in vitro*.

### 4.1. Consideration of throughput compared to published *in vitro* brain slice experiments

In drug discovery, the throughput performance of screening assays is of utmost importance. In this context, assays using cell lines with multielectrode array (MEA) measurements (Zhai et al., 2021) offer certain advantages. This method is also applicable to induced pluripotent stem (iPS) cells (Tukker et al., 2018, 2020). However, given the complex nature of seizure initiation, whole animal studies remain necessary. Brain slice experiments represent a step forward in increasing the throughput of seizure liability assays. More importantly, this method can improve our mechanistic understanding of seizures because the slice preparation provides access to the functioning of the neural network.

The throughput of brain slice experiments (Easter et al., 2007; Accardi et al., 2018; Zhai et al., 2021) is determined by the experimental procedures used to collect viable physiological tissue from the brain of a single animal. The time required to collect data is relatively consistent across experimental procedures. For example, in the study by Easter et al. (2007), it took approximately 70 min to collect data for a single compound (5 concentrations, 2 stimulus-response relationships), compared to 100 min in the present study, which included the stimulus-response relationship recordings. The VSD assay can visualize the range and extent of change in stimulus propagation, allowing quantitative analysis at any part of the recorded image field of view.

The active concentration ranges of the positive compound used in this study were compared with the active concentration ranges at which seizure liability assays (electrophysiology and MEA) using brain slices detected a seizurogenic response (Table 2). The concentrations of PiTX, GZ, and 4AP were considered to be within the range of existing assays. Pilo was not detected by the VSD-based assay, a point discussed further in section “4.3. Visualization of neural activity by VSD imaging.”

**TABLE 2:** Comparison with published *in vitro* seizure liability assay using brain slices.

Compound	Active concentration range (μM)		
	This study (VSD-based assay)	Published (assay method)	References
Picrotoxin	10–100	10–300 (PS number and PS area in EP)	Easter et al., 2007
		3–300 (PS number and PS area in EP)	Zhai et al., 2021
		0.2–100 (MEA)	Bradley et al., 2018
		10–100 (MEA)	Gao et al., 2017; Fan et al., 2019
Gabazine	1–10	1–10 (MEA)	Bradley et al., 2018
		0.3–3 (MEA)	Fan et al., 2019
4-aminopyridine	40–100	10–100 (PS number and PS area in EP)	Easter et al., 2007
		1–300 (PS number and PS area in EP)	Zhai et al., 2021
		11–100 (MEA)	Bradley et al., 2018
		10–100 (MEA)	Fan et al., 2019
Pilocarpine	ND	10–1000 (PS number and PS area in EP)	Zhai et al., 2021
		NEG (MEA)	Fan et al., 2019

EP, electrophysiology; MEA, multiwell microelectrode arrays or multi-electrode array; ND, not determined; NEG, negative for seizure liability.

### 4.2. fEPSP for monitoring the physiological response of the CA1 cortex

In the present study, we utilized fEPSP to monitor the slices’ physiological activity, as this method directly reflects synaptic activity. Seizure activity may be caused by various physiological processes in both presynaptic and postsynaptic systems. The changes induced by seizurogenic drugs should reflect alterations in synaptic causes, in addition to modifications in postsynaptic cells. In the electrophysiological assay, decreases in fEPSP slope were observed with gamma-aminobutyric acid type A (GABA_*A*_) receptor antagonists PiTX and GZ (Figures 2Aii, Bii), likely due to the intermission of a presynaptic GABAA receptor-mediated tonic facilitation of glutamatergic transmission (Jang et al., 2005). This may indicate the complex pathways of hippocampal disinhibition associated with GABA_*A*_ receptor antagonists (Bast et al., 2017). An increase in the fEPSP slope was observed with 4AP at a middle concentration (40 μM), as previously reported (Barish et al., 1996). However, 4AP showed no significant difference at a high concentration (100 μM) in contrast to a previous report (Wheeler et al., 1996). Although an increasing trend was observed at a high concentration (100 μM) of 4AP (Figure 2Cii), the cumulative increase of 4-AP dose concentration from low to high might explain this discrepancy. Additionally, a concentration-dependent decrease in fEPSP slope was observed with Pilo, whereas in a rat model of Pilo-induced temporal lobe epilepsy, a decrease in fEPSP slope was observed in the hippocampal slice after the onset of status epilepticus (Postnikova et al., 2021).

These observations highlight the synaptic changes caused by seizurogenic drugs. However, VSD imaging can provide access not only to the EPSP components at the stratum radiatum, along with the output function of principal cells at the stratum pyramidale (Figure 3), but also to the input-output function as the P-R ratio (Figure 5) and changes in different time windows (Figure 4: Immediate—Post-Immediate—Sustained ratios). This may reveal different mechanisms by which individual compounds induce seizures.

### 4.3. Visualization of neural activity by VSD imaging

Picrotoxin, GZ, and 4AP showed an expansion of the spread of neuronal activity in hippocampal slices and prolonged propagation time (Figure 3B), suggesting that a seizure-like response could be detected. The seizurogenic response of Pilo was not detected in this assay, nor could it be detected by multiwell microelectrode arrays (MEAs) using rat cortical neurons (Bradley et al., 2018). Similarly, MEAs using human induced pluripotent stem cell (hiPSC)-derived neuronal cells and rodent primary cortical cells did not detect dose-dependent and clear seizurogenic responses of Pilo (Tukker et al., 2020). Thus, detection of seizurogenic responses to Pilo could be challenging. However, VSD imaging using hippocampal slices from an animal model of Pilo-induced seizure demonstrated marked activation of the temporoammonic pathway (Ang et al., 2006). This suggests that depending on the drug, multiple pathways may need to be analyzed.

### 4.4. Exploiting the direct pharmacological impact on neuronal function using VSD assay parameters

In this study, we defined three parameters for the VSD response waveform: immediate peak, post-immediate peak, and sustained response (Figure 4). The VSD response waveform in the SR demonstrated an increase in sustained response, confirming a seizurogenic response for all compounds except Pilo (Figure 4E). For antagonists of the GABA_*A*_ receptor, increased sustained responses were observed with PiTX at high concentrations (100 μM) and GZ at middle or more concentrations (≥10 μM). The half-maximal inhibitory concentration (IC_50_) of PiTX for the GABA_*A*_ receptor is 1.15 μM (Ng et al., 2017), and that of GZ is 0.2 μM (Ueno et al., 1997). The difference in the active concentrations of seizurogenic responses was considered to be related to the difference in IC_50_ of the receptors. 4AP showed an increase in sustained response and ΔSPi (Figures 4Di, ii) at middle or high concentrations (≥40 μM). The application of 4-AP caused delayed depolarization following the initial synaptic response (Barish et al., 1996; Figure 4Di), whereas PiTX and GZ induced a prolonged depolarizing response (Figures 4Bi, Ci). This phenomenon is thought to be due to the inhibition of potassium current, a pharmacological effect of 4AP, and a similar phenomenon has been observed in rat brain slices (Barish et al., 1996).

We utilized the PR-ratio for evaluating the effects of PiTX, GZ, and 4AP. The PR-ratio reflects the E-S firing relationship and is calculated as the ratio of the amplitude at the SP to the SR (Tominaga et al., 2009). PiTX and GZ resulted in an increased PR-ratio (Figures 5Bii, Cii), suggesting that these compounds may induce a seizure response by reducing the shunting action of GABA receptors and lowering the threshold for spike firing (Mann and Paulsen, 2007). Similarly, 4AP exhibited an increased PR-ratio at low stimulation voltages (Figure 5Dii), indicating that it may also lower the threshold for spike firing and induce a seizure response. 4AP is known to inhibit D-type potassium conductance at low (<40 μM) concentrations (Storm, 1988) and inhibit A-type potassium conductance at higher concentrations (Gutman et al., 2005). These effects on potassium channels may affect the E-S firing function of the pyramidale cells and contribute to the modification of the PR-ratio. However, the PR-ratio of 4AP showed no significant change at 55-volt stimulation (Figure 5Dii), suggesting that delayed repolarization (Figures 4Di, ii) may induce a seizure response.

Overall, the parameters of the VSD assay, which directly reflect membrane potential responses, were useful in investigating the mode of action of unknown seizurogenic compounds by capturing their direct pharmacological impact on neuronal function. Our findings demonstrate the potential of VSD-based assays in identifying seizurogenic compounds during early drug discovery, thereby reducing delays in drug development and providing insights into the mechanisms underlying seizure induction and the associated risks of pro-convulsant compounds.

## Data availability statement

The original contributions presented in this study are included in the article/supplementary material, further inquiries can be directed to the corresponding author.

## Ethics statement

All animal experiments were performed according to protocols approved by the Animal Care and Use Committee of Tokushima Bunri University. The study was conducted in accordance with the local legislation and institutional requirements.

## Author contributions

YU and TT designed the research, analyzed the data, and wrote the manuscript. YU, MT, MM, YT, and TT performed the research. YT and TT developed the software. All authors contributed to the article and approved the final submitted version.
